# Surgical Fixation of a Comminuted Inter-Trochanteric Fracture in a Patient with Bilateral Below Knee Amputation

**DOI:** 10.5704/MOJ.1803.013

**Published:** 2018-03

**Authors:** BH Lee, SW Ho, CY Kau

**Affiliations:** Department of Orthopaedic Surgery, Tan Tock Seng Hospital, Singapore

**Keywords:** hip fracture, amputation, comminution, traction

## Abstract

Surgical fixation of hip fractures in patients with below knee amputation is challenging due to the difficulty in obtaining optimal traction for reduction of the fracture. Surgeons may face difficulty in positioning such patients on the traction table due to the absence of the foot and distal lower limb. There are several techniques described to overcome this technical difficulty. In this case report, we present a case of a 64-year old gentleman with bilateral below knee amputation presenting with a comminuted right intertrochanteric fracture. We highlight a simple and effective method of applying skin traction to obtain adequate reduction for hip fracture fixation.

## Introduction

With the advent of prosthetics, an increasing number of amputees are still ambulant and functionally active. When hip fractures occur in this group of patients, surgical treatment aims to optimise mobility and restore function. In surgical treatments of extra-capsular fractures, adequate reduction of the hip fracture is critical for proper fixation. Importantly, the positioning of the patient on the traction table is essential for a successful reduction. The patient’s foot on the affected side is secured to a boot attached to the traction table, which allows the surgeon to achieve adequate and stable control of traction and rotation for fracture reduction. However, the positioning of patients with distal lower limb amputations may pose a technical challenge. We present a case report of a 64-year old gentleman with bilateral below knee amputation who underwent a surgical fixation of a comminuted right intertrochanteric fracture. We discuss the methods described to overcome the technical difficulty and highlight a simple and effective method of applying skin traction to obtain adequate reduction for hip fracture fixation.

The authors have obtained the patient’s informed written consent for print and electronic publication of the case report.

## Case Report

**Fig. 1:**
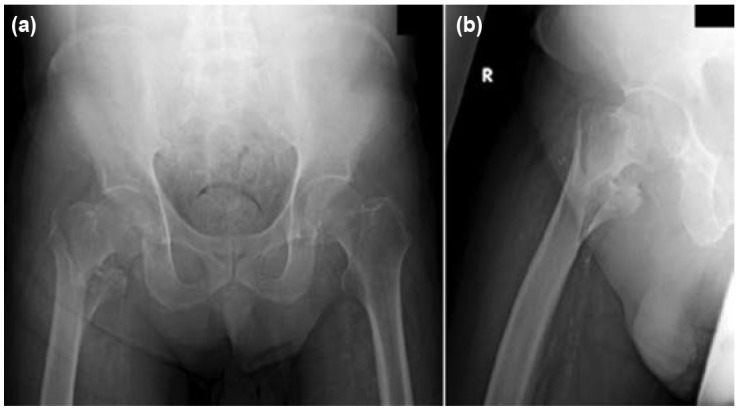
(a) Anterior-posterior view and (b) Lateral view of plain radiographs on admission showing a four-part displaced comminuted right intertrochanteric fracture.

A 64-year old gentleman presented to the emergency department in our institution with right hip pain and inability to weight bear following a fall. He had undergone bilateral below knee amputations due to peripheral vascular disease previously and was functionally ambulant with the use of bilateral below knee prostheses. Plain film radiographs revealed a displaced comminuted 4-part intertrochanteric fracture (Fig. 1). The patient was agreeable for surgical fixation of the hip fracture.

**Fig. 2:**
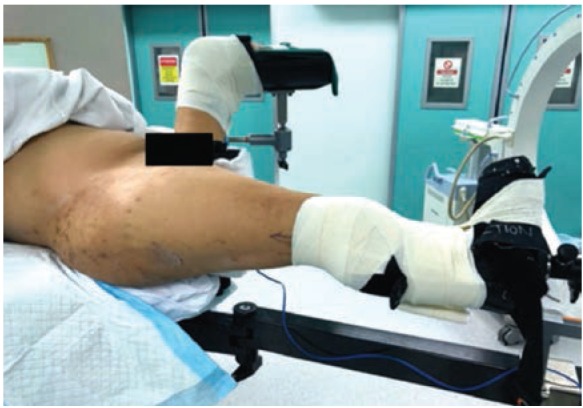
Positioning of patient on traction table. Strapping and securing of stump onto traction boot using elastic adhesive tape in “Figure-of-8” fashion circumferentially, around the boot to just proximal to the knee.

**Fig. 3:**
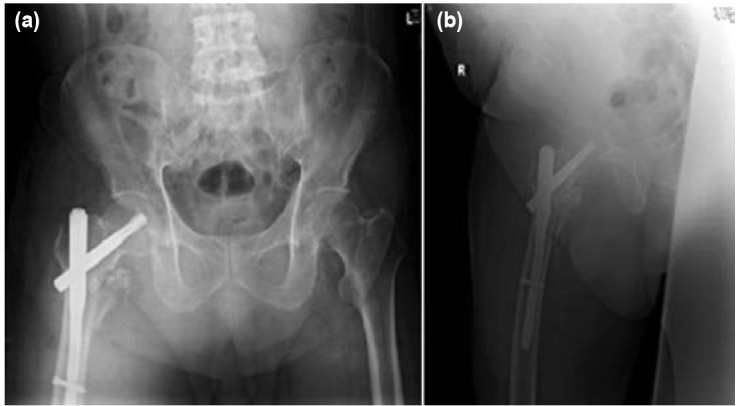
(a) Anterior-posterior view and (b) Lateral view of post-operative radiographs showing satisfactory reduction and fixation of fracture.

A standard fracture traction table (TenZor Carbon Fiber Traction Unit Assembly) was used to position the patient for intramedullary fixation. The below knee stump of the affected limb was strapped securely to the boot of the traction table via skin traction. Firstly, the distal stump was strapped to the boot of the traction table with the Velcro straps provided. Next, adhesive fabric tape [Elastic Adhesive Bandage, 10cm x 4.5cm, Smith & Nephew, UK] was used to secure the distal stump circumferentially in a “Figure-of-8” fashion, starting from the distal end of the boot, overlapping each layer by 50% of the width of the tape, all the way to 4.5cm proximal to the knee (width of the tape) (Fig. 2). The opposite below knee stump was placed in abduction to allow easy access for the image intensifier. This technique allowed for 25kg of traction to be applied as well as adequate internal rotation. Reduction manipulation was performed under guidance of image intensifier. Internal fixation was completed smoothly using a short statically locked intramedullary proximal femoral nail [Proximal Femoral Nail Antirotation, Depuy Synthes, USA]. The skin of the stump was inspected post-operatively and there were no open wounds, abrasions or skin lesions of concern. Postoperative radiographs were satisfactory (Fig. 3). The patient’s recovery was uneventful and he was discharged to a community hospital for further rehabilitation.

## Discussion

With the advancement of prosthetics, patients with bilateral lower limb amputations can still be functionally active. Hence, when hip fractures occur in these group of patients, operative intervention is a viable option to restore mobility and maximise their functional outcomes. Utilising a traction table for intertrochanteric fractures in below knee amputees poses a unique challenge as positioning the patient on the traction table is difficult due to absence distal part of the leg and foot. Proper positioning and traction is required to obtain reduction of the fracture and this is particularly critical when the fracture is comminuted and displaced.

Several techniques have been described in the literature to overcome this issue. The first is the use of skeletal traction for reduction of the fracture. This involves the insertion of Steinmann pin into the proximal tibia of the stump and an assistant’s help to position the affected limb for acceptable reduction^1^. An alternative is attaching the Steinman pin to the traction device. The disadvantages of this technique include the risks of infection, cutting out of the pin and soft tissue injury to the distal stump^1^. The inverted boot method, described by Nagesh *et al*^2^ utilises the fracture table by inverting the traction boot to accommodate the flexed knee and stump to provide traction and rotation control. The advantages of this technique were that fracture reduction and manipulation were effective and risks of skin injury and infection was reduced. However, there are some limitations to this method. The effectiveness of this technique is dependent on the length of the distal stump. There may be difficulty applying the boot in stumps less than 12cm^3^. In short stumps, the boot may obscure an unacceptable amount of the distal femur and this reduces the working surgical field, precluding the use of a long intramedullary nail by obstructing the insertion site of the distal interlocking screw. Where a long intramedullary nail is mandated, surgeons may have to resort to the insertion of a Steinmann pin in order to provide adequate traction as well as surgical exposure. The difficulty with applying the boot may be amplified in small sized patients with short femurs. In such cases, surgeons can consider utilising a paediatric foot traction boot which is compatible with the surgical table. Finally, the presence of a knee contracture may also pose difficulties in applying the boot as the stump should ideally be flexed to a 90 degrees angle for attachment.

Another method that has been described is the use of manual traction by an assistant to manipulate and maintain the fracture reduction or to fit the patient’s prosthesis to the boot of the traction table^3^. This technique is challenging as it is unlikely to provide satisfactory fracture reduction and control of the distal limb, especially in cases of displaced fractures, which often require a considerable amount of traction. In addition, manual traction by the assistant is difficult to maintain throughout the course of the surgery.

The technique described in this case report involves the use of skin traction to secure the stump to the traction boot. While skin traction techniques have been described in case reports, the fractures reported were mostly undisplaced or minimally displaced^4^. Rethnam *et al* suggested that when the fracture was displaced and greater traction anticipated, other techniques such as the inverted boot or skeletal traction should be utilised. It was thought that reduction manipulation and maintenance of fracture reduction was difficult with the use of skin traction^3^. In our experience, we have found that skin traction can still be used with satisfactory outcomes, even in cases of displaced and comminuted intertrochanteric fractures. In this case, the below knee stump of the affected limb was strapped securely to the boot of the traction table. We used adhesive fabric tape [Elastic Adhesive Bandage, 10cm x 4.5cm, Smith & Nephew, UK] to secure the distal stump circumferentially in a “Figure-of-8” fashion, around the boot, proximal to the knee. The Elastoplast is attached 4.5cm proximal to the knee (width of the tape) so as to maximise the surgical working field. By securing the knee joint, this provides not only a stable point of traction, but also allows for adequate rotation of the femur.

Davarinos *et al* noted that skin traction using adhesive fabric tape [Elastoplast 7.5cm x 4m, BSN Medical, UK] provided satisfactory adhesion and facilitated positioning of the limb. However, it provided limited rotational control^5^. In their technique, the adhesive tape was used to secure the limb in a circumferential manner. In our procedure, we did not encounter any technical issues with rotational control of the limb. This was probably due to our “Figure-of-8” technique using to secure the distal stump, rather than the type or brand of adhesive tape used.

Gamulin *et al* also proposed that a skin traction setup provides minimal rotation control and may be easily unravelled once a significant amount of traction was applied^1^. We did not experience such an issue with our technique. We were able to obtain up to 25kg of traction force utilising this method and were able to obtain satisfactory traction and rotation for fracture reduction. Moreover, this technique can still be used in the presence of a severe contracture in the knee joint, unlike the inverted boot technique. An important consideration when utilising this method is the skin condition. Surgeons should carefully assess the skin around the knee joint and stump prior to application of the elastic adhesive tape as any fragile areas may be prone to breaking down during the procedure. Given the ease of application and ability to provide adequate amounts of traction and rotation, surgeons should consider this method of skin traction when faced with obtaining traction for hip fractures in below knee amputees.

## Conclusion

Hip fracture reduction in a patient with below knee amputation poses a surgical challenge. Reduction with skin traction is an easy, effective and safe method in obtaining reduction for comminuted hip fractures in patients with below knee amputations.

## Conflict of Interest

The authors declare no conflicts of interest.
